# Users’ Willingness to Share Health Information in a Social Question-and-Answer Community: Cross-sectional Survey in China

**DOI:** 10.2196/26265

**Published:** 2021-03-30

**Authors:** PengFei Li, Lin Xu, TingTing Tang, Xiaoqian Wu, Cheng Huang

**Affiliations:** 1 College of Medical Informatics Chongqing Medical University Chongqing China; 2 Medical Data Science Academy Chongqing Medical University Chongqing China; 3 The Children’s Hospital of Chongqing Medical University Chongqing China

**Keywords:** health information, willingness to share information, structural equation model, Zhihu

## Abstract

**Background:**

Social question-and-answer communities play an increasingly important role in the dissemination of health information. It is important to identify influencing factors of user willingness to share health information to improve public health literacy.

**Objective:**

This study explored influencing factors of social question-and-answer community users who share health information to provide reference for the construction of a high-quality health information sharing community.

**Methods:**

A cross-sectional study was conducted through snowball sampling of 185 participants who are Zhihu users in China. A structural equation analysis was used to verify the interaction and influence of the strength between variables in the model. Hierarchical regression was also used to test the mediating effect in the model.

**Results:**

Altruism (β=.264, P<.001), intrinsic reward (β=.260, P=.03), self-efficacy (β=.468, P<.001), and community influence (β=.277, P=.003) had a positive effect on users’ willingness to share health information (WSHI). By contrast, extrinsic reward (β=−0.351, P<.001) had a negative effect. Self-efficacy also had a mediating effect (β=.147, 29.15%, 0.147/0.505) between community influence and WSHI.

**Conclusions:**

The findings suggest that users’ WSHI is influenced by many factors including altruism, self-efficacy, community influence, and intrinsic reward. Improving the social atmosphere of the platform is an effective method of encouraging users to share health information.

## Introduction

### Background

Social question-and-answer (Q&A) communities collect a large amount of high-quality health information based on the informal and collaborative method of information generation. Therefore, they have become an important means for the public to obtain health information. They also play an increasingly important role in promoting public health literacy. Zhihu is one of the most representative Q&A communities in China. In 2019, Zhihu had over 220 million registered users, over 28 million questions, and 130 million answers. On this platform, 750,000 questions were health-related, and nearly 21 million people followed the topic of health. In the Healthy China 2030 plan, the Chinese government requires news media to strengthen the publicity of health science knowledge. Moreover, news media is required to actively use social networks for health education. Therefore, exploring the influencing factors of users’ willingness to share health information (WSHI) based on the Q&A community is necessary and meaningful.

Users in a social Q&A community gather considerable amounts of high-quality health information through the sharing mechanism of question-answer-feedback. This topic has become one of the hot spots in medical informatics research to encourage more users to participate in producing health information. Empirically, Zhao et al [1] found that the interaction of intrinsic and extrinsic motivations has a considerable effect on users’ knowledge sharing willingness in a social Q&A community. He et al [2] updated the Open Access and Collaborative Consumer Health Vocabulary by mining user-generated health texts in such a social Q&A community to bridge the vocabulary gap between lay consumers and health care professionals. Exploration of the WSHI in a social Q&A community is the key to provision of appropriate services to users and an important guarantee for promotion of public health knowledge.

As public platforms, social Q&A communities were established on social networking sites (SNSs) for internet users to seek and share knowledge, experiences, and other information [3]. In a social Q&A community, users can ask or answer questions, comment on relevant content, agree or disagree with related views, and follow other users [4]. These features allow user information retrieval behavior not only by using keywords but also through the most direct form of asking questions about users’ complex information needs [5,6]. In terms of structure and function, the earliest social Q&A community is Quora. In this platform, users can ask their own questions and invite other users in corresponding fields to answer [5,7]. Zhihu is one of the most popular social Q&A platforms in China. It is often called the Chinese Quora. There are many similarities in the two platforms, such as user information exchange, content recommendation, and UI design. However, Zhihu and Quora have different development directions and operational concepts because they originate from different countries and social cultures. At present, Zhihu is China’s mainstream social Q&A community.

Thus far, the concept of WSHI has no unified definition. According to the self-determination theory proposed by Ryan et al [8], willingness is a psychological activity generated by an individual desire to perform a certain behavior based on various motivations. Health information sharing is one of the most important aspects in the research area of information sharing. Zhu et al [9] established an influencing factor model of patients’ willingness to share health information. The model includes variables of privacy concerns, online information support, information sensitivity, and disease severity. Abdelhamid et al [10] found that privacy concerns have the most influence on individuals’ intentions to share personal health information. Hah [11] analyzed health consumers’ health information–sharing behaviors from the perspective of the habit of using internet banking. On these bases, we define the WSHI as an individual psychological behavior driven by internal or external motivation. In the social Q&A community, such psychological behavior is often manifested as the willingness to ask health questions based on consumer experience or knowledge and provide health knowledge answers and express their views based on the content of the responses.

### Study Goal

The aim of this study is to establish a user WSHI model based on the social Q&A community environment. The study also seeks to explore factors that influence the sharing of health information among such users. There are many classical models in the area of research on health information sharing such as the social cognition theory [12], the theory of reasoned action [13], and the theory of planned behavior [14]. However, these classical theoretical models can only analyze users’ information-sharing behaviors from the perspective of psychological or social relations. The social Q&A community is an emerging online social platform, and the more complex information flow in such an environment warrants our more comprehensive consideration of this area. Furthermore, in consideration of the influence of community characteristics on users’ WSHI, it is necessary for one to establish a model suitable for the social Q&A community environment. This may be done by integrating various classical models. However, only a few studies put community influence into their models. Based on a structural equation, we attempted to bring the influences of community characteristics into this study. Meanwhile, we established a model of users’ WSHI in the social Q&A community environment and analyzed the influencing factors of the WSHI by verifying the proposed hypotheses in the model.

### Research Hypotheses

#### Altruism

Altruism is usually understood as an individual’s behavior of offering help to others at the expense of their own interests [15]. According to social exchange theory, we believe that altruism is a very complex psychological activity, and there are few behaviors that only consider others. From the perspective of social norms, altruism is a self-moral requirement based on individual ability and social influence [16]. Typically, the pleasant psychological feelings such as self-value perception and self-satisfaction are the pursuits of altruists [16]. Health information sharing is one of the behaviors that could help others solve their health problems and promote their health literacy. Therefore, altruists are more likely to identify with health information–sharing behaviors. Andrews et al [17] found that altruism is an effective factor for parents with children with genetic conditions so that these parents would share their child’s electronic health record. Obrenovic et al [14] separated tacit knowledge sharing from the scope of information sharing and found that altruism has a direct impact on tacit knowledge sharing. Lin et al [18] also suggested that altruism positively affects doctors’ attitudes toward knowledge sharing. On these bases, we propose hypothesis 1:

- H1: Altruism positively affects WSHI.

#### Intrinsic and Extrinsic Rewards

Intrinsic and extrinsic rewards are two of the most important concepts in social exchange theory [19,20]. In the study of WSHI, health information with social exchange value and the time and labor paid by individuals in these activities can be understood as a kind of commodity. Intrinsic and extrinsic rewards are the benefits that the users can expect to obtain after completing the commodity exchange. Health information can be regarded as a bargaining chip in a social exchange. Individuals can estimate how much they will be paid based on the health information they have shared. Social recognition such as respect and reputation are intrinsic rewards, whereas economic reward is an extrinsic reward. Researchers propose that intrinsic [21,22] and extrinsic rewards [23] are two of the main influencing factors in knowledge sharing. On these bases, we propose hypotheses 2 and 3:

- H2: Intrinsic reward positively affects WSHI.
- H3: Extrinsic reward positively affects WSHI.

#### Self-Efficacy

Self-efficacy refers to the subjective judgment of whether an individual can successfully implement a certain behavior and achieve the expected results in a specific environment and state [24]. This concept is derived from social cognition theory, which emphasizes the interaction among individual, behavior, and environment [12]. Self-efficacy is one of the most important individual factors in social cognition theory [12]. Kankanhalli et al [25] regarded self-efficacy as a factor for individuals to gain intrinsic benefits and believed that self-efficacy has a significant positive impact on users’ knowledge-sharing behavior. Kye et al [26] proposed in their empirical research that internet-related self-efficacy is positively related to information sharing. On this basis, we propose hypothesis 4:

- H4: Self-efficacy positively affects WSHI.

#### Community Influence

The characteristics of the community platform, such as design (Q&A format, agree/disagree mechanism, the *like* form, and comments), user stereotypes about the platform, impact of platform in this field, and protection of information sharers and their information can influence users’ WSHI in the social Q&A community. In this study, the features of the platform mentioned earlier are summarized as the variable community influence belonging to the category of objective variables of the influencing factors of user health information–sharing behavior. The improvement of community influence can reduce users’ perceptions of the difficulty of sharing health information, improve users’ self-efficacy, and promote actual sharing. On this basis, we propose the following hypotheses:

- H5a: Community influence positively affects user self-efficacy.
- H5b: Community influence positively affects WSHI.

## Methods

### Participant Selection

In this study, Zhihu users with a history of health information sharing were selected as the research objects. The sampling started with students at a medical university and extended by snowball sampling of an online questionnaire using WeChat and other message tools. This was done to maximize the population representation.

Zhihu users are mainly concentrated in the high knowledge-level population or people with a higher level educational background. Additionally, medical university students have high health literacy and will be the main health information disseminators in the future. Therefore, selecting medical university students as the starting point of snowball sampling is meaningful and necessary.

### Modeling

**Figure 1:**
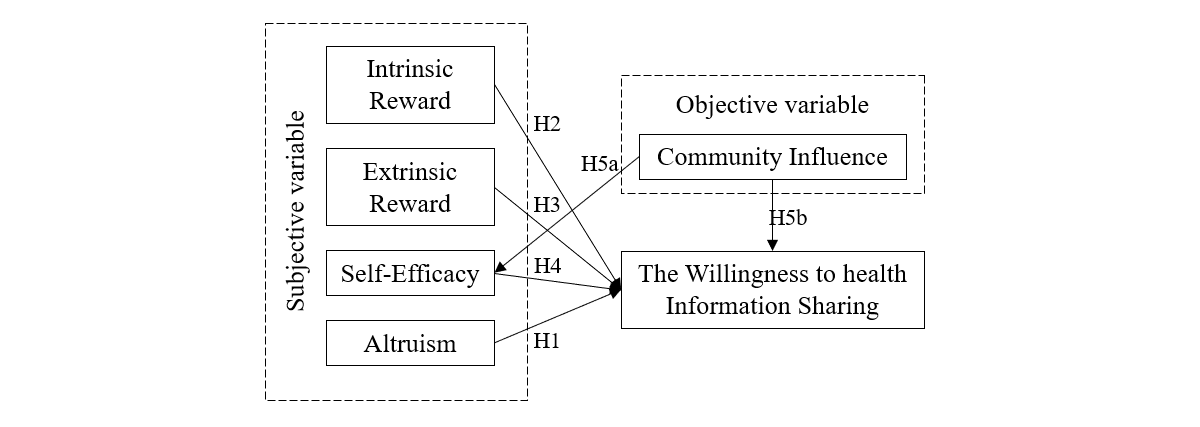
Willingness to share health information path model of the social question and answer community users.

This study explores the influencing factors of WSHI within the environment of a social Q&A community based on the interpretation of the above variables and related assumptions. Figure 1 illustrates the path model established from subjective and objective dimensions.

### Questionnaire

The observation indexes of the 6 variables in the model were screened according to the literature. A small-scale presurvey was conducted. Based on the results, the observation indexes of variables increased or decreased. Experts were asked to determine the questionnaire content in the form of a 5-point Likert scale. Table 1 lists the indexes and relevant explanations. The questionnaire is shown in Multimedia Appendix 1.

**Table 1:** Variables, indexes, and index descriptions in the model.

Variable, reference, and index description	
**Altruism—Lin [27]; Kankanhalli et al [25]**	
	AL1: I like to share my health information with other users on Zhihu.
	AL2: I think sharing health information on Zhihu can help others.
	AL3: I enjoy the process of helping others by sharing health knowledge on Zhihu.
	AL4: In my opinion, sharing health information on Zhihu is a manifestation of one’s social value.
**Intrinsic reward—Cho et al [28]; self-design**	
	IR1: I think by sharing health information on Zhihu, we can gain others’ respect.
	IR2: I think by sharing health information on Zhihu, I can gain praise and recognition from others.
	IR3: In my opinion, sharing health information on Zhihu can help me gain a more positive and confident attitude toward life.
**Extrinsic reward—Kankanhalli et al [25]; Cho et al [28]**	
	ER1: I think sharing health information can result in more followers.
	ER2: I think sharing health information on Zhihu can bring money or other material benefits.
**Community influence—self-design**	
	CI1: I think the Zhihu platform has high credibility in solving health problems.
	CI2: I think Zhihu is an important platform for me to obtain health information.
	CI3: I think the public image of Zhihu can promote users to share health information.
	CI4: I think Zhihu has certain security measures for sharers and the information they share.
	CI5: In my opinion, the platform design of Zhihu (question-and-answer format, agree/disagree mechanism, the *like* form, and comments) can promote one’s willingness to share health information.
**Self-efficacy—Lin [27]; Hsu et al [29]; Chen et al [30]**	
	SE1: I believe that the health information I have released on Zhihu is scientific and accurate.
	SE2: I can express my opinions on a topic on Zhihu with confidence.
	SE3: I can share new ideas and concepts about health information with others on Zhihu.
	SE4: I can provide rich content in other aspects for a certain health problem on Zhihu.
	SE5: I can accurately address the relevant issues and discuss them on Zhihu.
**Willingness to share health information—Schwarzer et al [31]; Bock et al [32]**	
	WSHI1: I am willing to share the health information I know on Zhihu.
	WSHI2: I would like to continue the practice of sharing health information.
	WSHI3: I will find more effective ways to share health information on Zhihu.
	WSHI4: I would like to participate in the discussion of health information content and express my views.
	WSHI5: I am willing to spend time to improve my knowledge system to provide others with better health information content.

### Data Collection and Exclusion

Zhihu users with a history of health information sharing were selected as the research objects. In this study, a history of health information–sharing behavior (screening criteria) was defined as follows:

- Publishing health-related information (including asking questions, answering questions, posting articles or ideas)
- Commenting on health-related information (20 words or more)
- Sharing or forwarding health-related information (eg, to WeChat, microblog, and other platforms)

The online questionnaire was issued from June 5 to June 19, 2020 (14 days). At the end of the period, 921 Zhihu user responses were collected. Among them, 210 users had previously shared health information. After eliminating responses with missing values, 185 valid responses were obtained. This number accounts for an effective rate of 88.10% (185/210).

### Statistical Analysis

Preprocessing, such as data filtering, was completed using Excel (Microsoft Corp) before importing information to the database. SPSS Statistics version 24.0 (IBM Corp) with AMOS version 24.0 and PROCESS [33] macro version 3.3 were used for data analysis. The continuous variables of demographic characteristics were classified. Subsequently, the frequency and percentage of each indicator were calculated. A structural equation analysis was used to verify the hypotheses and calculate the coefficients of each path in the model. PROCESS was used to verify whether the mediating effect between variables is significant. A P value not more than the test level set at .05 was considered to be statistically significant.

### Quality Control

The Cronbach alpha coefficient of the questionnaire was .961. It was well above .60 for each variable [34]. The composite reliability value was greater than 0.7 [35]. Table 2 presents details of the variables. All dimensions and the questionnaire as a whole have good internal consistency and reliability.

**Table 2:** Factor load, Cronbach alpha, average variance extracted, and composite reliability values of each variable.

Variable and index			Factor load		Cronbach alpha		AVE^a^		CR^b^
**Altruism**			—^c^		.875		.650		.881
	AL1	.754		—		—		—	
	AL2	.792		—		—		—	
	AL3	.906		—		—		—	
	AL4	.764		—		—		—	
**Community influence**			—		.894		.622		.892
	CI1	.806		—		—		—	
	CI2	.776		—		—		—	
	CI3	.797		—		—		—	
	CI4	.772		—		—		—	
	CI5	.792		—		—		—	
**Extrinsic reward**			—		.688		.638		.727
	ER1	.842		—		—		—	
	ER2	.672		—		—		—	
**Intrinsic reward**			—		.785		.755		.902
	IR1	.918		—		—		—	
	IR2	.922		—		—		—	
	IR3	.756		—		—		—	
**Self-efficacy**			—		.892		.628		.893
	SE1	.648		—		—		—	
	SE2	.848		—		—		—	
	SE3	.853		—		—		—	
	SE4	.776		—		—		—	
	SE5	.819		—		—		—	
**Willingness to share health information**			—		.928		.724		.929
	WS1	.787		—		—		—	
	WS2	.877		—		—		—	
	WS3	.850		—		—		—	
	WS4	.910		—		—		—	
	WS5	.824		—		—		—	

^a^AVE: Average variance extracted.

^b^CR: Critical ratio.

^c^Not applicable.

Content validity reflects the degree to which the description of measurement items affects the survey results. The measurement items in the questionnaire were mainly taken from the published literature. The self-designed indexes are obtained through expert discussion. They were then combined with the characteristics of the research object. Therefore, we believe that the scale has a good content validity. Structural validity includes both convergent and discriminant validities. The main measurement indexes are the factor load and average variance extracted (AVE) [35]. Table 2 presents the specific analysis results and index values. The factor loads and AVE values of all variables are greater than 0.5, indicating that the model has good convergent validity [35]. Discriminant validity requires the lowest possible correlation among all variables. Moreover, the standard is that such value should be less than the square root of the AVE value of the variable itself [36]. Table 3 indicates that the data on the diagonal are the square roots of the AVEs of each variable, indicating that the model has acceptable discriminant validity.

**Table 3:** Discriminant validity matrix.

Variable	AL^a^	CI^b^	ER^c^	IR^d^	SE^e^	WSHI^f^
AL	0.650	—^g^	—	—	—	—
CI	0.420	0.623	—	—	—	—
ER	0.619	0.628	0.638	—	—	—
IR	0.637	0.454	0.655	0.754	—	—
SE	0.562	0.261	0.505	0.721	0.628	—
WSHI	0.688	0.324	0.654	0.735	0.628	0.724
AVE^h^	0.806	0.789	0.799	0.868	0.793	0.851

^a^AL: Altruism.

^b^CI: Community influence.

^c^ER: Extrinsic reward.

^d^IR: Intrinsic reward.

^e^SE: Self-efficacy.

^f^WSHI: Willingness to share health information.

^g^Not applicable.

^h^AVE: Average variance extracted.

## Results

### Demographic Characteristics

Table 4 lists demographic characteristics of the participants. The data indicate that, among the participants, 70.8% (131/185) were female and 90.8% (168/185) were aged between 19 and 38 years. Additionally, 96.8% (179/185) had an undergraduate degree or higher education level. This survey considers medical students as the starting point of the snowball sampling considering the good educational background of Zhihu users and the fact that medical students will be the main producers and disseminators of health information in the future.

**Table 4:** Demographic characteristics of participants.

Variable		Value, n (%)
**Gender**		
	Male	54 (29.2)
	Female	131 (70.8)
**Age in years**		
	≤18	9 (4.9)
	19-38	168 (90.8)
	39-58	8 (4.3)
**Education**		
	Senior high school and below	3 (1.6)
	Junior college	3 (1.6)
	Undergraduate	143 (77.3)
	Master and above	36 (19.5)
**Background of majors**		
	Medical science or related	139 (75.1)
	Nonmedical-related	46 (24.9)
**Profession**		
	Student	150 (81.1)
	Government personnel	11 (5.9)
	Professional technical personnel	11 (5.9)
	Business and service personnel	4 (2.2)
	Other	9 (4.9)

### Model Test

Table 5 presents the path and model fitting using the SPSS Statistics 24.0 and AMOS 24.0 software. We selected the chi-square/degree of freedom (χ^2^/df), root mean square error of approximation (RMSEA), incremental fit index (IFI), and cumulative fit index (CFI) as reference indexes of the model fitting. When χ^2^/df < 3, RMSEA < 0.08 and IFI/TLI/CFI > 0.9, the model is considered to have a good fit [37-39]. Table 5 indicates that χ^2^/df = 1.95 < 3 and RMSEA = 0.072 < 0.08. Other fitting indexes are all above 0.9. Therefore, the model fit is acceptable.

**Table 5:** Model fitting test index values.

Index	Value	Standard	Fitting
*χ*^2^/df^a^	1.959	<5	acceptable
		<3	ideal
RMSEA^b^	0.072	<0.08	acceptable
		<0.05	ideal
IFI^c^	0.934	＞0.9	ideal
CFI^d^	0.933	＞0.9	ideal

^a^*χ*^2^/df: Chi-square/degree of freedom.

^b^RMSEA: Root mean square error of approximation.

^c^IFI: Incremental fit index.

^d^CFI: Cumulative fit index.

**Figure 2:**
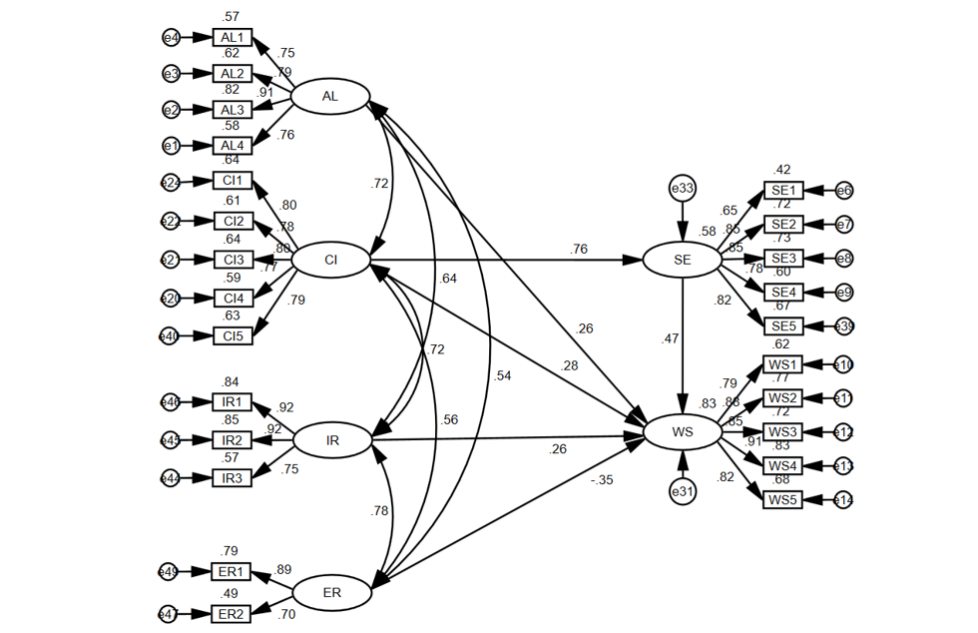
Structural equation model path diagram. AL: altruism; CI: community influence; IR intrinsic reward; ER: extrinsic reward; SE: self-efficacy; WS: willingness to share.

Figure 2 is the path diagram of the structural equation model (SEM). By contrast, Table 6 lists the path coefficients. The influence path of each variable on health information–sharing intention is significant. Altruism, community influence, intrinsic reward, and self-efficacy all positively affect WSHI. According to the absolute value of the influence of independent variables on dependent variables, the ranking is as follows: self-efficacy (β=.468; P<.001), extrinsic reward (β=−.351; P<.001), community influence (β=.277; P=.003), altruism (β=.264; P<.001), and intrinsic reward (β=.260; P=.03). All of the hypotheses (H1, H2, H4, H5a, and H5b) are true except for H3.

**Table 6:** Path testing.

Path	Unstandardized coefficient	Standardized coefficient	Standard error	CR^a^	P value
CI^b^→SE^c^	0.814	.764	0.092	8.818	<.001
SE→WSHI^d^	0.520	.468	0.095	5.313	<.001
AL^e^→WSHI	0.285	.264	0.087	3.446	<.001
CI→WSHI	0.328	.277	0.125	2.963	.003
IR^f^→WSHI	0.291	.260	0.153	2.157	.03
ER^g^→WSHI	–0.347	–.351	0.101	–3.433	<.001

^a^CR: Critical ratio.

^b^CI: Community influence.

^c^SE: Self-efficacy.

^d^WSHI: Willingness to share health information.

^e^AL: Altruism.

^f^IR: Intrinsic reward.

^g^ER: Extrinsic reward.

### Mediating Effect Test

Based on the PROCESS [33] macro, we tested the mediating effect of self-efficacy through hierarchical regression. The study selected a bootstrap to sample 2000 times and set altruism, extrinsic reward, and intrinsic reward as control variables to test the mediating effect of self-efficacy in the influence of community influence on WSHI. Table 7 indicates that the SEM is significant (Δ*r*^2^=.063, Δ*F*=5.305, P<.001). The first line in Table 7 corresponds to the dependent variables of model 1, model 2, and model 3, respectively. The difference between model 2 and model 3 is whether the mediating effect of self-efficacy is introduced. The *r*^2^ and F score values for these three models were as follows: model 1 (*r*^2^=.521, F=49.019), model 2 (*r*^2^=.651, F=83.874), and model 3 (*r*^2^=.714, F=89.179). As shown in Table 8, in the path community influence → self-efficacy → willingness to share, the mediating effect value of self-efficacy was 0.147. Moreover, the effect accounted for 29.15%. The bootstrap test indicated that the 95% confidence interval did not contain 0. Therefore, the mediating effect was significant.

**Table 7:** Model for testing the mediating effect of self-efficacy.

Variable	SE^a^		WSHI1^b, ^^c^			WSHI2^d^	
	*t* score	P value	*t* score	P value	*t* score		P value
AL^e^	2.387	.02	5.169	<.001	4.506		<.001
ER^f^	0.609	.54	–2.298	.02	–2.811		.006
IR^g^	3.102	.002	3.883	<.001	2.754		.007
CI^h^	4.680	<.001	6.804	<.001	5.012		<.001
SE	—^i^	—	—	—	6.261		<.001

^a^SE: Self-efficacy.

^b^WSHI: Willingness to share health information.

^c^No Moderating variables.

^d^Self-efficacy was introduced as a moderating variable.

^e^AL: Altruism.

^f^ER: Extrinsic reward.

^g^IR: Intrinsic reward.

^h^CI: Community influence.

^i^Not applicable.

**Table 8:** Proportion of the mediating effect.

Mediating effect	β	Boot SE	Boot upper	Boot lower	%
Total	.505	0.088	0.327	0.670	—
Direction	.358	0.082	0.198	0.517	70.83
Mediation	.147	0.047	0.063	0.245	29.15

## Discussion

### Principal Findings

#### Influence of Altruism on Willingness to Share Health Information

Altruism has a positive effect on the user’s WSHI. From the perspective of social norms, altruism is a moral requirement and standard for individual social values based on one’s ability and social influence. In other words, this is the self-perception of “with great power comes great responsibility.” In a social Q&A community, users tend to exert certain moral requirements and restrictions on themselves based on their own cognitive ability and knowledge. These include the sharing of the health information they know and grasping to help other community users. Raj et al [40] suggested that altruism is one of the important factors that promote the moral obligation of individuals to share health information for research. In a social Q&A community, a good social atmosphere can help users achieve more in-depth communication with others and maximize user exposure to health information needs. This finding, that optimizing the social atmosphere is an effective measure, is of considerable significance for generating altruistic psychology among users. To a certain extent, the discussion atmosphere in the community can be optimized through filtering of users and strict auditing of users’ content publishing.

#### Influence of Intrinsic and Extrinsic Rewards on Willingness to Share Health Information

In the context of a social Q&A community, intrinsic reward has a positive effect on WSHI, but extrinsic reward has a negative impact. Users typically respect, praise, and thank the information sharers when users improve their health with the help of information shared by others. Intrinsic reward, such as respect and reputation, can promote a sense of satisfaction, pleasure, and fulfillment among health information sharers. In turn, this state of mind can continue to generate their WSHI. Thus, intrinsic reward (ie, reputation) can positively affect users’ willingness in health information sharing [18,23]. To further improve users’ perceptions of intrinsic reward, the community should, on the premise of ensuring that spam information is effectively filtered, magnify the exposure of other users to the effective feedback content of relevant health information. This may be done by means of group chat and push. Similarly, increasing intrinsic reward entails ensuring that the magnification of this exposure is known to the health information sharer in the social Q&A community. Another necessity is an effective 2-way mutual evaluation function in which health information recipients can rate or express opinions on the sharers and their shared information. Moreover, the health information sharers should also be able to rate the opinions of recipients. At the same time, rewards (ie, membership points, experience value, and level promotion) are given based on the mutual recognition of both parties.

The conclusion that extrinsic reward negatively affects users’ willingness in health information sharing differs from that of existing research. This finding may be caused by the demographic distribution characteristics of the study’s current sample. The participants are mainly college students with their family or parents as their main sources of income. After the lower economic pressure is mapped to these users and their WSHI, it was found that intrinsic reward (ie, reputation and respect) can have a greater influence than extrinsic reward. Conversely, inappropriate extrinsic reward may cause user aversion or resistance. The users may feel that their sharing behavior is controlled by the organization if extrinsic reward is the intention of sharing health information. Just like the imposition of punishment from the organization, material reward is another mechanism of controlling individual behavior. This can cause an individual to lose interest and enthusiasm in sharing health information [41]. Tamir et al [42] found that individuals are willing to forgo money to share their experiences and knowledge. However, given the demographic distribution characteristics of the current samples, we do not deny that some professional web writers, who spend more time online and have more followers, will benefit from the flow economy by sharing health information. Therefore, it is necessary to further subdivide health information–sharing users to obtain more realistic and objective results. Meanwhile, the specific mechanism through which extrinsic reward influences WSHI also needs further study.

#### Effect of Community Influence and Self-Efficacy on Willingness in Health Information Sharing

Community influence and self-efficacy have a positive effect on users’ WSHI. Moreover, self-efficacy has a mediating effect similar to the way that community influence, an objective variable in the environment, affects WSHI. This objective fact includes various aspects such as community development philosophy, platform design, information protection, and user group influence. These factors interact with each other, and they not only have a direct impact on the WSHI but also exert further influence by positively affecting users’ sense of self-efficacy. The perception and evaluation of factors such as self-ability and environmental conditions are necessary steps before users share health information. Users tend to be satisfied with their information-sharing behavior in perceiving that their own knowledge can help other users [43]. Improving the design of the community platform and strengthening the information protection mechanism and publicity efforts of the community can attract more high-quality users to participate in sharing health information. This will in turn improve the overall influence of the community. At the same time, we should also pay attention to the effect of community influence on user self-efficacy. After realizing the accurate identification and tagging of social Q&A community users, it can push the needs of health information demanders to health information providers more efficiently through a reasonable information push mechanism. This shall stimulate the generation of user self-efficacy. Thus, the virtuous cycle of health information diffusion is effectively promoted.

### Strengths and Limitations

Many scholars have conducted in-depth research in the field of knowledge sharing. They proposed different knowledge sharing models for different types of information or communication environments [44,45]. However, most of the relevant studies did not consider the impact of the characteristics of these information dissemination environments on users’ willingness to share knowledge. This study attempted to bring influences of community characteristics into the model of users’ WSHI. As a result, we found that the variable community influence has a positive impact on users’ WSHI. Meanwhile, the variable self-efficacy has a mediating effect between community influence and users’ WSHI. This study provides new ideas and directions in the research area of users’ WSHI and proposes suggestions on promoting users to share health information. These suggestions can be used as a reference for health information service providers to formulate health intervention strategies.

This study also has certain limitations. First, only Zhihu users were taken as the objects of this research. Thus, not all social Q&A community users are covered. Follow-up research should further improve the coverage of social Q&A community users. Second, the samples mainly comprised ordinary Zhihu users, and key users with a large number of followers are underrepresented. Third, although the sample population, mainly comprising medical college students, can better represent the information-sharing behavior of the general population, the snowball sampling method used in this study still has systematic errors. Fourth, the method of using an online questionnaire may introduce bias of demographic characteristics. Finally, the data obtained were formed by subjective reports provided by participants. Overly conservative or exaggerated choices can lead to a certain degree of bias in the statistical results. More scientific experimental designs can be adopted to avoid the biases caused by these deficiencies in follow-up studies. Despite these shortcomings, this study presents novel ideas, and the results provide new insights into the promotion of WSHI.

### Conclusions

Promoting the dissemination of high-quality health information is important for guiding users of a social Q&A community to actively participate in health information sharing. Compared with relevant research, this study introduces the variable community influence into the model based on the characteristics of a social Q&A community. Additionally, combining the variables intrinsic reward, extrinsic reward, altruism, and self-efficacy, WSHI’s SEM in a social Q&A community is constructed. The results indicate that intrinsic reward, altruism, and self-efficacy have a positive effect on WSHI. By contrast, extrinsic reward has a negative effect. Self-efficacy has a mediating effect on the relationship between community influence and WSHI. The generation of WSHI may be promoted by paying more attention to the social atmosphere of the community, optimizing the gratitude feedback mechanism, and striving to build good social relations among users. The results can provide a theoretical and practical reference for social Q&A community operators, health education and promotion, and other aspects.

## Appendix

Multimedia Appendix 1 Questionnaire.

## Abbreviations

AVE: average variance extracted
CFI: cumulative fit index
IFI: incremental fit index
Q&A: question-and-answer
RMSEA: root mean square error of approximation
SEM: structural equation model
SNS: social network site
WSHI: willingness to share health information
χ2/df: chi-square/degree of freedom
