# Supplementary material for: Users’ Willingness to Share Health Information in a Social Question-and-Answer Community: Cross-sectional Survey in China
Source: JMIR Med Inform. 2021 Mar 30;9(3):e26265. doi: 10.2196/26265 (PMC8075348; doi:10.2196/26265)
Supplement: Multimedia Appendix 1 [file medinform_v9i3e26265_app1.docx]

No._____________

**Questionnaire on the Willingness of Social Question-And-Answer Community Users to Share Health Information**

Dear Sir/Madam：

Good days!

This is a questionnaire about Zhihu users' willingness to share health information (such as hair loss, staying up late, information, experience and knowledge of COVID-19, etc.). If you have ever used The Zhihu question-and-answer community, we would be grateful if you could use a few minutes to complete the following questionnaire. The research group promises that all your personal answers will only be used for academic research. Please feel free to fill in.

Your serious answer is of great significance to this study. Please fill it out truthfully. Thank you again for your cooperation!

Instructions:

1 Please do not fill in or mention your name in the questionnaire;

2 There is no right or wrong answer, just check it according to your own situation;

3 Please fill in the blanks with your answers at the horizontal line;

4 Please use "√" to check the corresponding option that matches your actual situation.

**Personal Information**

1. gender

①male ②female

2. Age

①≤18 ②19~38 ③39~58 ④59~68

⑤69 and above

3 education

①senior High school and Below ②junior college

③undergraduate ④master and above

4 Background of majors

①medical science or related majors ②non-medical related majors

5 profession

①students

②the government personnel

③professional technical personnel

④business and service personnel

⑤Agricultural production personnel

⑥Production and transportation equipment operators and related personnel

⑦soldier

⑧the others

6 Have you ever used Zhihu

①YES

②NO（Stop answering）

7 The way you come into contact with Zhihu is（Multiple choice）

①Search engine（such as google, Baidu, etc.）

②WeChat ③Microblog

④TV ⑤APP Store

⑥through friends

⑦others：________

8 The health information (such as information, experience and knowledge about pneumonia epidemic, hair loss, staying up late, diet, health maintenance, etc.) you have done on Zhihu include（Multiple choice）

①publish health-related information (including asking questions, answering questions, Posting articles or ideas, etc.).

②comment on health-related information (20 words or more).

③Search for health-related information.

④Click a like for health-related information.

⑤share or forward health-related information (e.g. to WeChat, Microblog, etc.).

⑥Browse for health-related information.

⑦Collect health-related information.

⑧others：________

9 Please evaluate your health level (including physical, mental and social health)

①poor ②medium ③good ④Excellent

**Survey on the willingness to share health information on Zhihu**

Please tick "√" in the serial number corresponding to your situation according to your understanding and usage habits.

1: Not at all. → 5: It fits perfectly.

| Item | Score | | | | |
| --- | --- | --- | --- | --- | --- |
|  | 1 | 2 | 3 | 4 | 5 |
| AL1: I like to share my health information with other users on Zhihu. |  |  |  |  |  |
| AL2: I think sharing health information on Zhihu can help others. |  |  |  |  |  |
| AL3: I enjoy the process of helping others by sharing health knowledge on Zhihu. |  |  |  |  |  |
| AL4: In my opinion, sharing health information on Zhihu is a manifestation of one's social value. |  |  |  |  |  |
|  | | | | | |
| IR1: I think by sharing health information on Zhihu, we can gain others' respect. |  |  |  |  |  |
| IR2: I think sharing health information on Zhihu can gain praise and recognition from others. |  |  |  |  |  |
| IR3: In my opinion, sharing health information on Zhihu can help me gain a more positive and confident attitude towards life. |  |  |  |  |  |
|  | | | | | |
| ER1: I think sharing health information can result in more followers. |  |  |  |  |  |
| ER2: I think sharing health information on Zhihu can bring money or other material benefits. |  |  |  |  |  |
|  | | | | | |
| CI1: I think the Zhihu platform has high credibility in solving health problems. |  |  |  |  |  |
| CI2: I think Zhihu is an important platform for me to obtain health information. |  |  |  |  |  |
| CI3: I think the public image of Zhihu can promote users to share health information. |  |  |  |  |  |
| CI4: I think Zhihu has certain security measures for sharers and the information they share. |  |  |  |  |  |
| CI5: In my opinion, the platform design of Zhihu (Q&A format, agree/disagree mechanism, “like” form, comments) can promote the willingness to share health information. |  |  |  |  |  |
|  | | | | | |
| SE1: I believe that the health information I have released on Zhihu is scientific and accurate. |  |  |  |  |  |
| SE2: I can express my opinions on a topic in Zhihu with confidence. |  |  |  |  |  |
| SE3: I can share new ideas and concepts about health information with others on Zhihu. |  |  |  |  |  |
| SE4: I can provide rich content in other aspects for a certain health problem on Zhihu. |  |  |  |  |  |
| SE5: I can accurately cut into the relevant issues and discuss them on Zhihu. |  |  |  |  |  |
|  | | | | | |
| WSHI1: I am willing to share the health information I know on Zhihu. |  |  |  |  |  |
| WSHI2: I would like to continue the practice of sharing health information. |  |  |  |  |  |
| WSHI3: I will find more effective ways to share health information on Zhihu. |  |  |  |  |  |
| WSHI4: I would like to participate in the discussion of health information content and express my views. |  |  |  |  |  |
| WSHI5: I am willing to spend time to improve my knowledge system to provide others with better health information content. |  |  |  |  |  |

**The questionnaires are all over. Thank you again for your cooperation！**
